# Carbonic Anhydrase Inhibitors as Novel Drugs against Mycobacterial β-Carbonic Anhydrases: An Update on *In Vitro* and *In Vivo* Studies

**DOI:** 10.3390/molecules23112911

**Published:** 2018-11-08

**Authors:** Ashok Aspatwar, Jean-Yves Winum, Fabrizio Carta, Claudiu T. Supuran, Milka Hammaren, Mataleena Parikka, Seppo Parkkila

**Affiliations:** 1 Faculty of Medicine and Health Technology, University of Tampere, 33014 Tampere, Finland; milka.hammaren@staff.uta.fi (M.H.); mataleena.parikka@staff.uta.fi (M.P.); seppo.parkkila@staff.uta.fi (S.P.); 2 Institut des Biomolécules Max Mousseron (IBMM) UMR 5247 CNRS, ENSCM, Université de Montpellier, 34296 Montpellier CEDEX 05, France; Jean-yves.winum@umontpellier.fr; 3 Neurofarba Department, Sezione di Chimica Farmaceutica e Nutraceutica, Università degli Studi di Firenze, 50019 Sesto Fiorentino (Firenze), Italy; fabrizio.carta@unifi.it (F.C.); claudiu.supuran@unifi.it (C.T.S.); 4 Oral and Maxillofacial Unit, Tampere University Hospital, 33521 Tampere, Finland; 5 Fimlab Ltd. and Tampere University Hospital, 33520 Tampere, Finland

**Keywords:** mycobacterial diseases, β-carbonic anhydrases, *Mycobacterium tuberculosis*, drug targets, carbonic anhydrase inhibitors, in vivo inhibition, *in vitro* inhibition

## Abstract

Mycobacteria cause a variety of diseases, such as tuberculosis, leprosy, and opportunistic diseases in immunocompromised people. The treatment of these diseases is problematic, necessitating the development of novel treatment strategies. Recently, β-carbonic anhydrases (β-CAs) have emerged as potential drug targets in mycobacteria. The genomes of mycobacteria encode for three β-CAs that have been cloned and characterized from *Mycobacterium tuberculosis* (Mtb) and the crystal structures of two of the enzymes have been determined. Different classes of inhibitor molecules against Mtb β-CAs have subsequently been designed and have been shown to inhibit these mycobacterial enzymes *in vitro*. The inhibition of these centrally important mycobacterial enzymes leads to reduced growth of mycobacteria, lower virulence, and impaired biofilm formation. Thus, the inhibition of β-CAs could be a novel approach for developing drugs against the severe diseases caused by pathogenic mycobacteria. In the present article, we review the data related to *in vitro* and *in vivo* inhibition studies in the field.

## 1. Introduction

Mycobacteria are rod-shaped, non-motile, and acid-fast bacteria that contain a high amount of G + C in their genome [1,2]. The genus mycobacterium includes a variety of clinically relevant human pathogens, including *Mycobacterium tuberculosis* (Mtb), the main causative agent of human tuberculosis (TB). A group of closely related bacteria referred to as the Mycobacterium tuberculosis complex (MTC) composes of a variety of pathogens causing TB in humans and other mammals. These include *M. tuberculosis*, *M. bovis*, *M. caprae*, *M. africanum*, *M. canettii, M. microti*, *M. orygis*, and *M. pinnipedii* [3]. Mtb is typically transmitted through air by a droplet contact. Mtb can affect many organs in humans, but the main target organ is the lung, causing pulmonary TB in 80% of the patients. In 2017, 10 million new cases were diagnosed [4]. In addition to the diagnosed active Mtb infections, the World Health Organization (WHO) estimates that 23% of the world’s population has developed a latent TB [4], which is asymptomatic but can become reactivated and cause a difficult-to-treat and potentially lethal disease. Mtb is currently one of the deadliest bacteria killing 1.3 million people every year. Multi-drug-resistant strains of Mtb are on the rise making TB increasingly difficult to treat. This development poses an enormous global threat necessitating immediate action to find new ways to treat this devastating disease [4].

Leprosy is another example of a clinically relevant mycobacterial disease. Leprosy is caused by *M. leprae*, which is transmitted through droplets due to close and frequent contact with untreated patients. As the multiplication rate of *M. leprae* is very slow, the incubation period of disease ranges between 1 and 20 years. The disease mainly affects the skin, peripheral nerves, mucosa of the upper respiratory tract, and eye. If left untreated, the disease usually causes permanent tissue damage. In many developing countries, leprosy is still a serious health problem and the people suffering from the disease often face social problems that go hand in hand with the disease progression. The latest WHO report shows that there were 216,108 new leprosy cases in 145 countries from the 6 WHO regions [5].

The nontuberculous mycobacteria (NTM) group includes all mycobacteria other than MTC and *M. leprae*, and about 40 species of them are pathogenic [6]. NTM are ubiquitously found in a wide variety of environmental reservoirs [7,8]. Although they are mostly nonpathogenic, they are important opportunistic pathogens of humans [9]. The species of NTM associated with human disease are: *M. avium, M. intracellulare*, *M. kansasii*, *M. fortuitum*, *M. chelonae*, *M. szulgai*, *M. paratuberculosis*, *M. scrofulaceum* [10] as well as bacteria belonging to the *M. abscessus* complex [11]. NTM can cause pulmonary disease resembling tuberculosis, lymphadenitis, and skin disease. The pulmonary disease represents about 80% of infections caused by NTM [12]. Recent reports suggest that the NTM pulmonary disease is increasing in several parts of the world [13,14]. However, standardized diagnostics and effective treatment protocols for NTM infections are lacking [15].

Genomes of many mycobacterial species from both MTC and NTM categories have been sequenced [16,17,18,19,20]. Bioinformatic and molecular analysis of mycobacterial genomes revealed that they code for several novel proteins that are essential for the alternative pathways and critical for the life cycle of these pathogens [21,22,23]. Recent progress in the structural and functional analyses of genomes and proteomes has opened new avenues for the design of mechanism-based drugs targeting proteins crucial for pathogenesis of mycobacteria [24]. Among many such proteins, β-carbonic anhydrases (β-CAs) of mycobacteria could be possible targets for developing novel antimycobacterial agents with the potential to treat even infections caused by drug-resistant mycobacteria.

**Figure 1:**
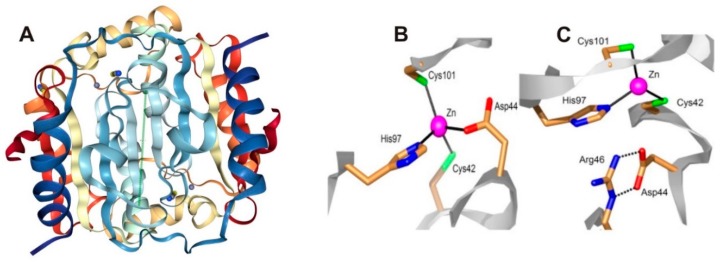
Crystal Structure of Rv1284 (β-CA1) from *M. tuberculosis*. (**A**) Structure of β-CA1 (1YLK) [22]. Coordination of the Zn(II) ion in the β-CA1 of Mtb. (**B**) Closed active site, with the Zn(II) ion (violet sphere) coordinated by a histidine, two cysteines and one aspartate residue. (**C**) Open active site, with three protein ligands coordinated to Zn(II); the aspartate makes a salt bridge with a conserved arginine residue in all β-CAs [22,29]. The images adapted from Covarrubias et al. https://www.rcsb.org/structure/1YLK [22]. Licensed under CC BY 4.0.

*M. tuberculosis* genome codes for three β-CA genes Rv1284 (β-CA1), Rv3588c (β-CA2) and Rv3273 (β-CA3) as shown in Table 1 [25]. Database searches and our bioinformatic analyses showed the presence of all the three β-CAs in both NTM and MTC bacteria [26,27,28,29,30]. β-CAs catalyze the reversible hydration of CO_2_ to HCO_3_^−^ and H^+^, thus generating a buffering weak base (bicarbonate) and a strong acid (H^+^) [31,32]. Mycobacterial β-CAs are zinc-containing metalloenzymes with characteristics similar to many other bacterial β-CAs. All conserved amino acid residues typical of β-CAs and involved in the catalytic cycle, i.e., the four zinc-binding residues, Cys42, Asp44, His97 and Cys101 are shown in Figure 1.

The mycobacterial β-CAs are essential during starvation for the growth and survival of the bacteria [22,23,33,34]. Recent studies showed that the bicarbonate ion, which is a product of reversible hydration of CO_2_, is essential for the transport of extracellular DNA (eDNA) and the formation of biofilm in NTM bacteria *in vitro* [35]. Inhibition of β-CAs using ethoxzolamide (EZA), a CA inhibitor, reduced the transport of eDNA and the formation of biofilm [35]. EZA also inhibited the PhoPR regulon, a two-component regulatory system in Mtb, as well as Esx-1 protein secretion system centrally important for the virulence of Mtb bacterium, and showed efficacy in infected macrophages and mice [36], suggesting that β-CAs perform very important roles in mycobacterial infections. Using *M. marinum*, an NTM model bacterium, we were the first to show that dithiocarbamate Fc14-584b, a β-CA inhibitor impairs mycobacterial growth in zebrafish larvae *in vivo* [26]. These essential enzymes are thus potential drug targets and are currently under investigation by several groups, including ours [26,27,28,29,35,36,37,38,39]. Similarly, several *in vitro* studies have shown that all the Mtb β-CAs could be efficiently (*K_I_* in nanomolar ranges) inhibited by sulfonamides/sulfamates (Table 1). In the present review, we update the data on *in vitro* and *in vivo* studies using CA inhibitors on mycobacterial β-CAs.

## 2. *In Vitro* Inhibition Studies of *M. tuberculosis* β-CAs

### 2.1. Sulfonamides as Inhibitors of M. tuberculosis β-CAs

**Figure 2:**
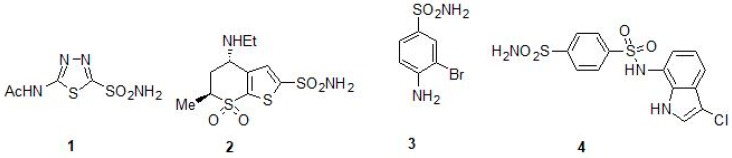
Chemical structures of acetazolamide (**1**) (ATZ), dorzolamide (**2**) (DZA), 3-bromosulfanilamide (**3**) and indisulam (**4**).

The cloning and characterization of the *M. tuberculosis* β-CAs were done in the 2000s and these enzymes were identified as novel drug targets for developing anti-TB agents [27,28,29]. β-CA1 was the first Mtb β-CA cloned and characterized and in the same study, the first *in vitro* inhibition studies were performed using a panel of sulfonamides, sulfamates and their derivatives [27]. For *in vitro* inhibition studies, the CO_2_ hydration activity of β-CA1 was measured by applying Applied Photophysics stopped-flow instrument using phenol red as an indicator [27]. Among the tested sulfonamides, most of them inhibited the activity of β-CA1 in the range of 1–10 µM. Many of the derivatives, including sulfanilyl-sulfonamides acetazolamide (ATZ) (1), methazolamide, dichlorophenamide, dorzolamide (DZA) (2), brinzolamide, benzolamide, and the sulfamate topiramate, exhibited sub-micromolar inhibition (*K_I_* values of 0.481–0.905 µM) [27] (Table 2). Among the tested sulfonamides 3-bromosulfanilamide (3) and indisulam (4) inhibited the activity of β-CAs most efficiently (*K_I_* values of 97–186 nM) (Table 2 and Figure 2). This was the first study to show that Mtb β-CA1 is a potential target for developing anti-TB drugs that have a different mechanism of action [27]. Several studies *in vitro* inhibition studies were performed using these inhibitor molecules on Mtb β-CA1 and human CA II that showed similar inhibition profiles suggesting reliability of the method used for the studies [29,39,40,41,42,43,44,45].

**Figure 3:**
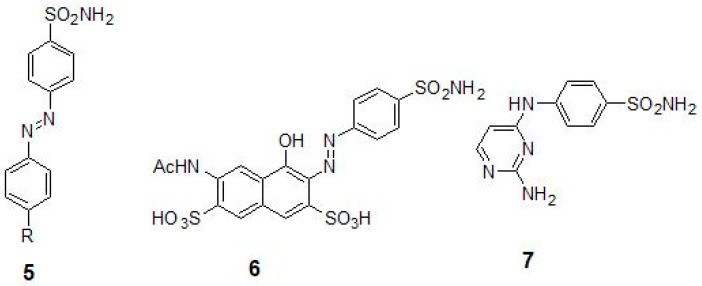
Chemical structures of diazenylbenzenesulfonamides (**5**), prontosil (**6**) and 2-amino-pyrimidin-4-yl-sulfanilamide (**7**).

Inhibition of Mtb β-CA2 was investigated using a series of diazenylbenzenesulfonamides (5) that were derived from sulfanilamide or metanilamide (Table 2) [40]. To increase the inhibitory properties, new molecules were synthesized by diazotization of aminosulfonamide and by coupling with phenols or amines [29]. The molecules were subsequently incorporated with various R moieties in the molecule such as hydroxy, amino, methylamino and dimethylamino and sulfonate that may induce water solubility to these compounds as sodium salts. The aminomethylene sodium sulfonate derivatives and their corresponding *N*-methylated analogue showed the best inhibition constants (*K_I_*s of 45–59 nM) [29]. In these compounds, the para position had bulky substituent with respect to the sulfonyl moiety, suggesting that this strategy may be good in obtaining low nanomolar range inhibitors that are selective against Mtb β-CA2 [29]. In addition to Mtb β-CA1 and β-CA2, the diazenylbenzenesulfonamides (5) were also tested for the inhibition of the Mtb β-CA3, and the prontosil (6) (Table 2 and Figure 3) was found to be the best inhibitor with inhibition constants in the range of (*K_I_*s) of 126–148 nM [39]. In another study, several compounds were studied for their inhibitory properties against Mtb β-CA3 and among them 2-amino-pyrimidin-4-yl-sulfanilamide (7) (*K_I_* 90 nM) and sulfonylated sulfonamide (*K_I_* of 170 nM) showed that Mtb CA3 can be successfully targeted using CAIs with a potential for developing agents targeting mycobacteria (Table 2) [28].

**Figure 4:**
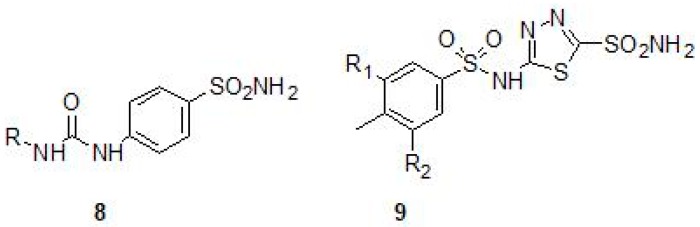
General chemical structures of ureido containing sulfonamides (**8**) and halogenated benzene sulfonamides (**9**).

Inhibition studies on Mtb β-CA1 and β-CA3 using sulfonamides prepared by reaction of sulfanilamide with aryl/alkyl isocyanates (Ureido-sulfonamides) (8) have been carried out and the *K_I_*s were found to be in the range of 4.8–6500 nM and of 6.4–6850 nM, respectively (Table 2) [41]. Similarly, inhibition studies on all the three β-CAs of Mtb were performed using a number of halogenated sulfanilamides and halogenated benzolamide (9) derivatives that showed the efficacies of inhibition in the sub-micromolar to micromolar range (Table 2 and Figure 4). The inhibition range was dependent on the substitution pattern at the sulfanilamide moiety/fragment of the molecule. Best inhibitors were the halogenated benzolamides (*K_I_*s in the range of 0.12–0.45 μM), whereas the halogenated sulfanilamides were slightly less inhibitory (*K_I_*s in the range of 0.41–4.74 μM) [42].

**Figure 5:**
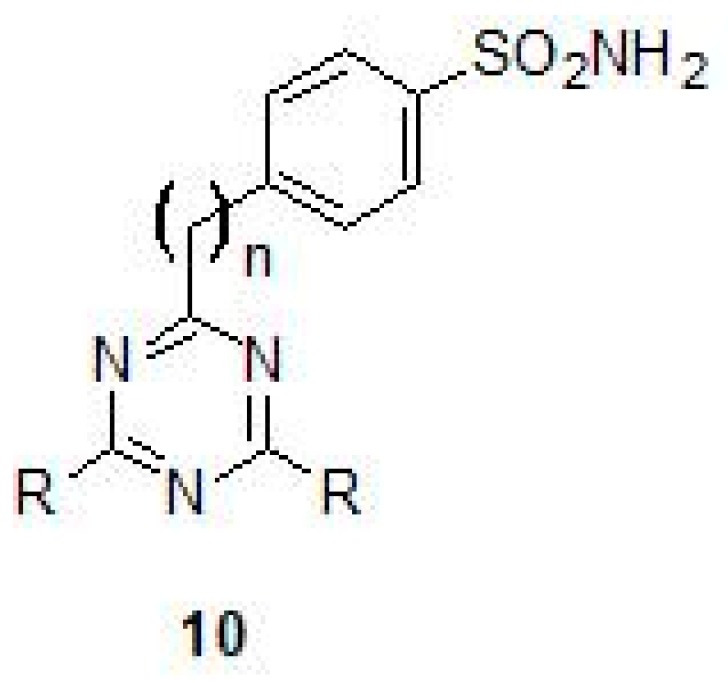
General chemical structure of triazinyl sulfonamides (**10**).

A new series of fluorine containing sulfonamides (Triazinyl sulfonamides) (10) that were incorporated with amino, amino alcohol and amino acid moieties were used for the inhibition of all the three β-CAs of Mtb [43] (Figure 5). Among the compounds tested, some of them inhibited β-CA2 efficiently with *K_I_* values in the nanomolar range and also with very good potency (*K_I_*s in sub-micromolar range) against β-CA1 and β-CA3 [43] (Table 2). In a recent study, novel sulfonamides were obtained from sulfanilamide, which was N4-alkylated with ethyl bromoacetate, followed by reaction with hydrazine hydrate and further reacted with various aromatic aldehydes [44]. The inhibition studies using these sulfonamides showed *K_I_*s in the range of 127 nM–2.12 μM for Mtb β-CA3 [44].

### 2.2. Mono and Dithiocarbamates

**Figure 6:**
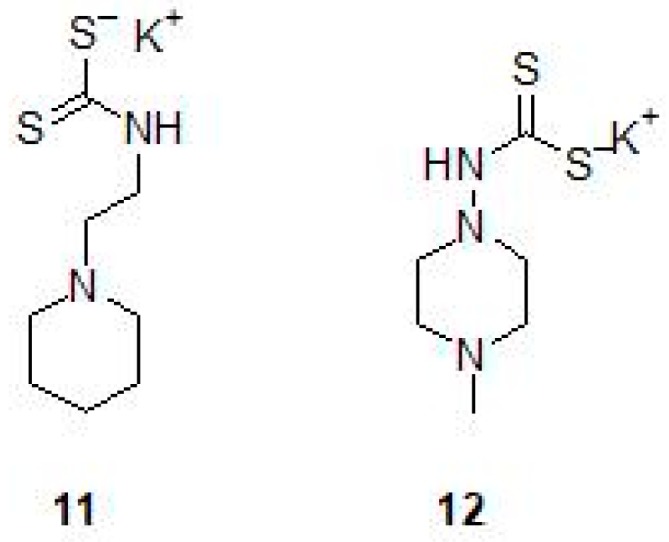
Chemical structures of dithiocarbamates (**11**) and (**12**).

A series of *N*-mono- and *N*,*N*-disubstituted dithiocarbamates (DTCs) (11,12) have been tested for inhibition of β-CA1 and β-CA3 from Mtb (Table 2 and Figure 6) [45]. Both enzymes could be inhibited with sub-nanomolar to micromolar efficacies, depending on the substitution pattern at the nitrogen atom from the dithiocarbamate zinc-binding group. Aryl, arylalkyl-, heterocyclic as well as aliphatic and amino acyl moieties led to potent Mtb β-CA1 and β-CA3 inhibitors in both the *N*-mono- and *N*,*N*-disubstituted dithiocarbamate series [45].

### 2.3. Phenolic Natural Products and Phenolic Acids

Several studies have shown that sulfonamides inhibit the Mtb β-CAs efficiently as discussed above. Similarly, in an effort to discover novel inhibitors that could selectively inhibit β-CAs through novel mechanism of action, Supuran’s group screened a series of phenolic-based natural products (NPs) against the Mtb β-CAs [46]. Enzyme inhibition properties of 21 NP compounds were investigated against β-CAs of Mtb as well as against human α-CAs I and II for comparison. Sulfonamides that are used clinically inhibited human CAs efficiently (at nM range), whereas β-CAs required micromolar concentrations. In contrast, 8 and 7 of the 21 phenolic compounds had sub-micromolar affinity for β-CA1 and β-CA3, respectively. The selectivity of some compounds was significantly higher against β-CAs than human α-CAs (the inhibition range being 8 μM to 430 μM) [46]. These NPs are the first nonclassical CA inhibitors that are more potent against mycobacteria β-CAs compared to host CA enzymes, suggesting usefulness of NPs for targeting β-CA of Mtb [46]. In addition to natural phenolic products, a series of phenolic acids and their esters, derivatives of caffeic, ferulic, and p-coumaric acids were tested against all the β-CAs of Mtb [47]. Among the screened compounds, esters 6–9 showed good inhibitory activity against all the Mtb β-CAs (*K_I_*s 1.87 Μm–7.05 μM), whereas they showed no inhibitory activity against human CAI and CAII, suggesting that they could be potentially developed as anti-mycobacterial compounds [47]. Computational analysis of binding mode of the compounds suggested that the inhibitors anchor to the zinc-coordinated water molecule from the CA active site interfering with the nucleophilic attack of the zinc hydroxide on the substrate CO_2_ [47]. These results provided insights into mechanism of inhibition of β-CAs, which may be valuable for developing new mycobacterial agents with a novel mechanism of action [47].

**Figure 7:**
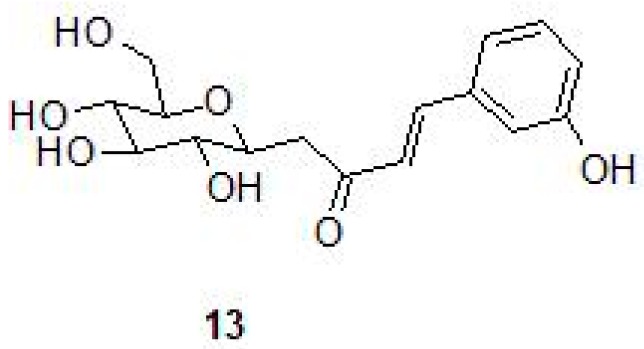
Chemical structure of *C*-cinnamoyl glycoside (**13**).

In another study, inhibition profiles of series of *C*-cinnamoyl glycosides (13) containing the phenol moiety were investigated against the three β-CAs of Mtb [48] (Table 2 and Figure 7). Among the compounds investigated, most of them (compounds **1**–**3** and **5**–**7**) inhibited Mtb β-CA2 at nanomolar concentrations (*K_I_* 130-640 nM), and for Mtb β-CA1 (compounds **5** and **6**) the *K_I_* range was between 140–930 nM and showed preference for β-CA1 over human CAII. Only one compound inhibited Mtb β-CA1 at nanomolar quantities (*K_I_* 140 nM) over human CAII [48].

### 2.4. Carboxylic Acids

**Figure 8:**
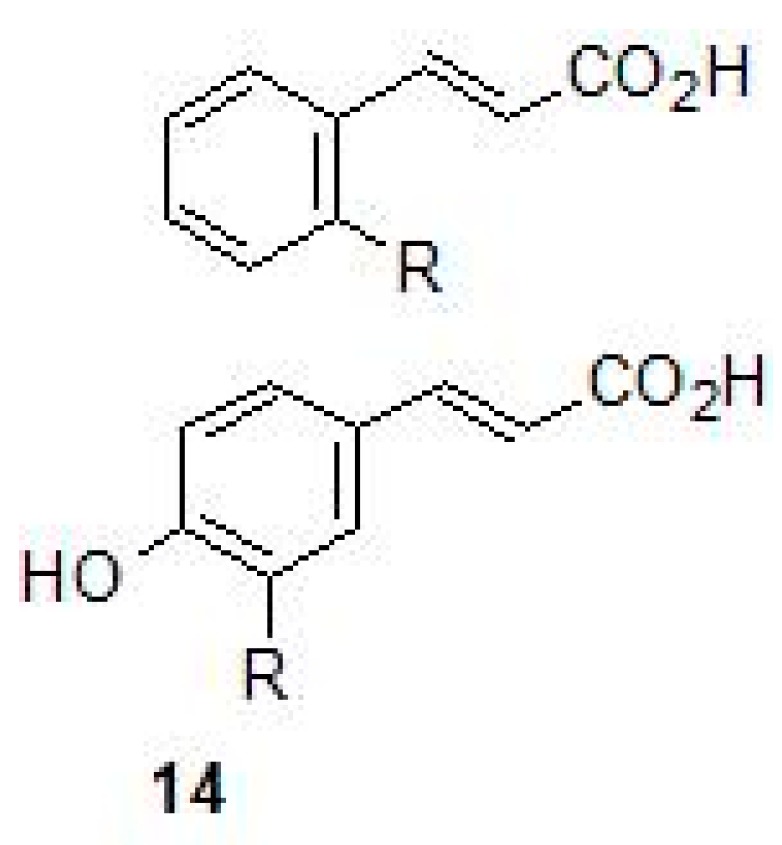
General chemical structures ferulic and coumaric acids (**14**).

Weak acids are known to inhibit the growth of mycobacterium but the mechanism of action of these compounds is not known. Carboxylic acids (14) that contain scaffolds such as benzoic acids, nipecotic acid, ortho and para coumaric acid and ferulic acid were investigated for the inhibition of all the three β-CAs of Mtb (Figure 8). These compounds inhibited all the three β-CA enzymes of Mtb at sub-micromolar to micromolar concentration range (*K_I_*s in the range of 0.11–0.97 µM). The *K_I_*s for the inhibition of β-CA2 was in the range of 0.59–8.10 µM, whereas against β-CA1, the carboxylic acids showed inhibition constants in the range of 2.25–7.13 µM [49]. This class of relatively underexplored β-CA inhibitors warrant further in vivo studies, as they may have the potential for developing antimycobacterial agents.

## 3. *In Vitro* Inhibition of *Mycobacterial strains* Using CA Inhibitors

A new class of compounds prepared by reaction of 6-mercaptopurine with sulfony/sulfenyl halides known as 9-sulfonylated/sulfenylated-6-mercaptopurines inhibit growth of Mtb H37Rv, a wild type bacilli in the range of 0.39–3.39 μg/mL [50] (Table 3). In addition, one of the derivatives showed an appreciable (minimal inhibitory concentration (MIC) under 1 μg/mL) inhibitory activity against several drug resistant strains of Mtb [50]. The compounds that exhibit MIC of less than 1 μg/mL are considered as excellent leads and were the first CAIs with anti-tubercular activity. Thus, these compounds may indeed constitute interesting leads for discovering more efficient antimycobacterial drugs. Similarly, *C*-cinnamoyl glycosides containing the phenol moiety that inhibit Mtb β-CA1 and β-CA2 in nanomolar quantities were tested for inhibition of the Mtb H_37_Rv strain, leading to the identification of compounds having anti-tubercular activity (Table 3) [48]. The MIC of the *C*-cinnamoyl glycosides was 100 μg/mL; though high, the compounds inhibited the growth of the bacterium completely. Interestingly, one of the *C*-cinnamoyl glycosides, (*E*)-1-(2,3,4,6-tetra-*O*-acetyl-β-d-glucopyranosyl)-4-(3-hydroxyphenyl) but-3-en-2-one inhibited the growth of the bacterium efficiently (3.125–6.25 μg/mL) on a solid medium (Table 3) [48].

Inhibition studies on *M. marinum,* an NTM and a close relative of Mtb, were carried out in liquid cultures using DTCs Fc14-584b and Fc14-594a. These drugs were prepared by reaction of corresponding amine with carbon disulfide in the presence of a base and shown to be specific inhibitors of Mtb β-CA1 and β-CA3 [45]. *In vitro* inhibition studies showed that the concentration required for the inhibition of the *M. marinum* was 17–18 μg/mL for both compounds after six days of exposure to the inhibitors. Further studies to find if the compounds were bacteriostatic or bactericidal showed that there was no growth resumption of *M*. *marinum* with inhibitor concentration below MIC after inhibitor dilution by 1:4, suggesting that these compounds were bactericidal [26] (Table 3).

**Figure 9:**
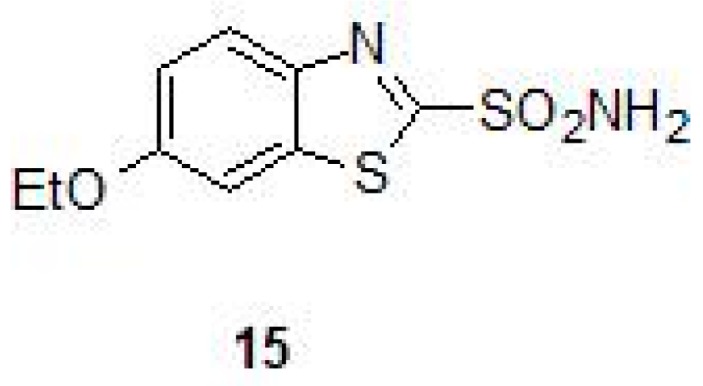
Chemical structure of ethoxzolamide (**15**).

Similar to other bacteria that contain extracellular DNA (eDNA) in the matrix of the bacterial biofilms, NTM bacteria also contain significant amounts of eDNA in their biofilms and are responsible for phenotypic resistance of the bacteria to antibiotics, in addition to other biological functions [51]. A recent study showed that bicarbonate ion positively influences eDNA export in NTM and it is well established that bicarbonate is generated by the hydration of carbon dioxide via CA [31,35]. Screening of a mutant library for eDNA export in NTM bacteria *M. avium* identified mutants that were inactivated for *CA* gene and these mutants when complemented with the *CA* gene restored the transport of eDNA, suggesting that CAs play important roles in the transport of eDNA and formation of biofilms in NTM [35]. The surface exposed proteome of *M. avium* in eDNA containing biofilms showed presence of abundant CAs and inhibition studies exposing these bacteria to 6-ethoxy-1,3-benzathiazole-2-sulfonamide/ethoxzolamide (**15**) (EZA) showed reduction in eDNA transport significantly (Table 4 and Figure 9) [35]. Thus, in addition to having an effect on mycobacterial growth, CA-inhibition may also be a potential strategy to inhibit biofilm formation of mycobacteria.

## 4. CA Inhibitors and *In Vivo* Inhibition of Mycobacteria

**Figure 10:**
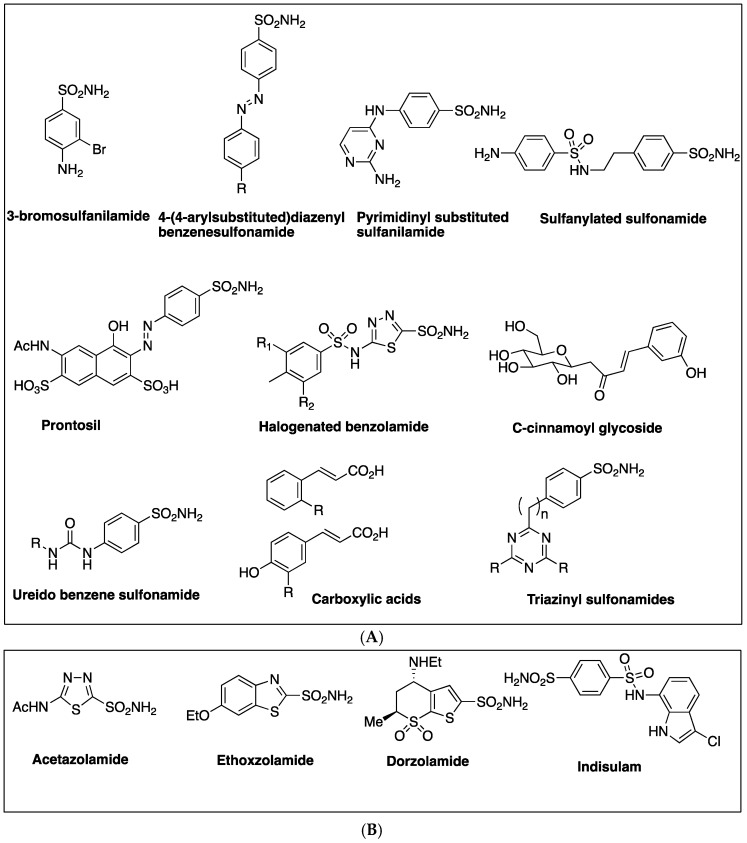
Novel compounds that inhibit *M. tuberculosis* β-CAs at nanomolar quantities. (**Upper panel**; **A**): structures of the compounds with a potential to be developed as anti-mycobacterial agents for treating the mycobacterial diseases caused by MTC and NTM bacteria that are resistant to clinically used drugs. (**Lower panel**; **B**): compounds that efficiently inhibit the *M. tuberculosis* β-CAs in addition to human CAII and are in clinical use or testing to treat human diseases.

The first *in vivo* study to show the effect of CA inhibitor on Mtb was published in 2015 by Johnson et al. [36]. The authors showed that EZA (Figure 10) inhibits the signaling of PhoPR in Mtb [36,52]. EZA is a sulfonamide compound (Figure 10) that is a general inhibitor of CA enzyme activity and is an FDA approved drug used in the treatment of glaucoma, epilepsy and duodenal ulcers and is a diuretic.

The study showed that Mtb treated with EZA induces phenotypes similar to the mutants of the PhoPR, downregulating PhoPR regulon, reducing the production of virulence-associated lipids, and inhibiting Esx-1 protein secretion (Table 4) [36]. In addition, quantitative single cell imaging of a PhoPR dependent fluorescent reporter strain showed that EZA inhibits PhoPR regulated genes in infected macrophages and mouse lungs [36]. Similarly, the efficacy assessment in *Mtb*-infected mice, orally treated with EZA, showed a significant reduction in bacterial growth in the lungs compared to the mock-treated control group [36].

**Figure 11:**
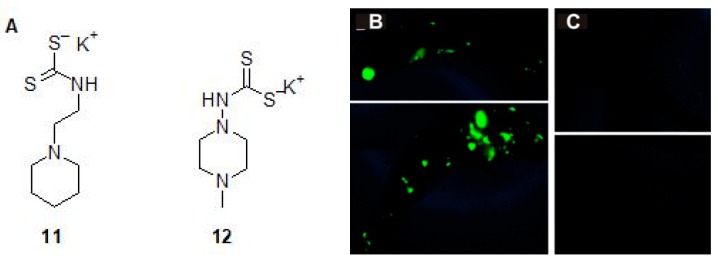
The *in vivo* inhibition of *M. marinum* in zebrafish larval model. (**A**) The β-CA inhibitors dithiocarbamate 11 and 12 that were evaluated for toxicity and safety for further use to inhibit growth of *M. marinum* in zebrafish larvae. (**B**) The zebrafish larvae injected with *M. marinum* wasabi strain, which expresses a green fluorescent protein. The green fluorescence showing the infection in zebrafish larvae after 6-day post infection not treated with any inhibitor. (**C**) Zebrafish larvae injected with *M. marinum* and treated with 300 μM concentration of 12 [26].

Dithiocarbamates, another class of compounds that strongly inhibit β-CAs of Mtb *in vitro* have been recently used by our group to show *in vivo* inhibition of *M. marinum* [26,45]. Among the two DTCs **11** and **12** that were first evaluated for toxicity in the zebrafish larval model, **12** was found to be less toxic and was taken further to study the inhibition of *M. marinum in vivo*. The zebrafish larvae infected with green fluorescent *M. marinum* strain with an infection dose of 471 ± 143 bacteria treated with 300 μM concentration of **12** showed significant reduction (*p* > 0.0096) in bacterial load compared to the larvae not treated with the inhibitor (Figure 11C). The study suggested that the inhibitors of CAs could be useful as a new class of antimycobacterial compounds that can potentially treat MDR-TB.

## 5. Future Prospects

In this review, we discussed the progress made on the discovery and development of antimycobacterial agents that target mycobacterial β-CAs. The chemical inhibitors that selectively bind to the mycobacterial β-CAs could be developed as antimycobacterial agents for treating not only drug-resistant tuberculosis, but also other diseases caused by pathogenic mycobacteria that are resistant to clinically used drugs. The *M. tuberculosis* contains three β-CAs, among them, β-CA1 and β-CA 2 are cytoplasmic, and β-CA3 is membrane associated. Many of the inhibitors reported so far have been shown to inhibit β-CA1 and β-CA2 *in vitro* efficiently, however it is not known if these inhibitors are permeable through the mycobacterial membrane. Similarly, many of the inhibitors that have been shown to inhibit mycobacterial β-CAs efficiently have also been shown to inhibit human α-CAs though in higher concentrations.

The current strategy of developing inhibitors against the mycobacterial β-CAs for treating mycobacterial diseases can be more successful in the future by designing inhibitors that bind Mtb β-CAs selectively and specifically. For designing such inhibitors, information regarding the active site residues of the enzymes that interact with inhibitor molecules need to be obtained. Resolving crystal structures in complex with potential inhibitors of β-CAs is one way of getting insights into such residues that will help in the design and synthesis of β-CA specific inhibitors.

Studies have shown that these enzymes can be inhibited *in vivo* using CAIs exhibiting antimycobacterial effect showing proof-of-concept. However, only very few studies have shown antimycobacterial effects of the inhibitors possibly through the inhibition of β-CA3 of the bacterium. In future, to achieve more success for *in vivo* inhibition of these enzymes, there is a need to design and synthesize inhibitors that are not only selective for β-CAs but also permeable through the membrane. It will also be useful to design inhibitor molecules with a tag that will help in tracking the fate of the molecule once it is inside the bacterium.

Zebrafish represents an excellent vertebrate model for tuberculosis research, because it is a natural host for *M. marinum* that causes a TB-like disease in the fish. Safety and toxicity of the potential β-CA inhibitors can be first evaluated using zebrafish larvae. Subsequently, preclinical *in vivo* inhibition studies can be done in a zebrafish larval model after causing an active TB by *M. marinum* infection. The recent developments of the *in vivo* zebrafish models that mimic the human TB disease, coupled with new imaging technologies, provide much better predictive preclinical models to produce new combinations of treatments/drugs that are more effective against the hard-to-treat mycobacterial diseases. Unlike the drugs that are in clinical use, the β-CA inhibitors have a different mechanism of action. These drugs will probably have minimal off-target effects due to the absence of β-CAs in humans and other vertebrates in whom mycobacteria cause infections.

## Figures and Tables

**Table 1:** Activity and inhibition properties of Mtb β-CAs compared to human CA II.

CAs	Gene ID	Protein	*K*_cat_ (s^−1^)	*K*_cat_/Km (M^−1^ s^−1^)	Activity	*K*_I_ (nM) ^a^	Reference
Mtb β-CA1	Rv1284	163 aa	3.9 × 10^5^	3.7 × 10^7^	Moderate	480	[27]
Mtb β-CA2	Rv3588c	207 aa	9.8 × 10^5^	9.3 × 10^7^	High	9.8	[29]
Mtb β-CA3	Rv3273	^b^ 215 aa	4.3 × 10^5^	4.0 × 10^7^	Moderate	104	[28]
hCAII	*CA2*	260 aa	1.4 × 10^6^	1.5 × 10^8^	Very high	12	[29]

^a^ Inhibition using acetazolamide. ^b^ CA domain of β-CA3.

**Table 2:** Compounds that inhibit *M. tuberculosis* β-CAs at nM concentrations compared to inhibition of human CAII enzyme *in vitro*.

CAIs	hCAII	β-CA1	β-CA2	β-CA3	Reference
3-bromosulfanilamide	40	186	NS	NS	[27]
Diazenylbenzenesulfonamides	105	-	45–955	-	[29]
2-amino-pyrimidin-4-yl-sulfanilamide	33	-	NA	91	[28]
Sulfonylated sulfonamide	NS	-	-	170	[28]
prontosil	NS	126		148	[39]
Halogenated benzolamides	NS	120–580	410–450	170–340	[42]
*N*-mono- and *N*,*N*-dithiocarbamates	0.7–325	0.9–481	NA	0.91–431	[45]
Ureido-sulfonamides	2.1–226	5–560	NA	6.4–533	[41]
Cinnamoyl glycosides	NS	140	130–640	-	[48]
Triazinyl Sulfonamides	4.9–5.5	42–580	8.1–10	2.1–210	[43]
^a^ Acetazolamide (ATZ)	12	481	9	104	[29]
^a^ Ethoxzolamide (EZA)	8	-	594	27	[29]
^a^ Dorzolamide (DZA)	9	744	99	137	[29]
^a^ Indisulam	15	97	NS	NS	[27]

^a^ Clinically, the most relevant or promising compounds that inhibit human CAs efficiently. NA—not assayed, NS–non-significant.

**Table 3:** Studies on minimal inhibitory concentrations (MICs) of the CA inhibitors in mycobacterial cultures.

Inhibitor	Bacilli	Concentration	Reference
(*E*)-1-(2,3,4,6-tetra-*O*-acetyl-β-d-glucopyranosyl)-4-(3-hydroxyphenyl) but-3-en-2-one	Mtb H_37_Rv	^b^ 3.125–6.25 μg/mL	[48]
		100 μg/mL	[48]
DTCs (Fc14-584b and Fc14-594a)	*M. marinum*	17–18 μg/mL	[26]
9-sulfonylated/sulfenylated-6-mercaptopurines	Mtb H_37_Rv	0.39–3.39 μg/mL	[50]
9-sulfonylated/sulfenylated-6-mercaptopurines	^a^ Mtb	1 μg/mL	[50]

^a^ Drug resistant Mtb strains. ^b^ Antimycobacterial activity on solid medium.

**Table 4:** Details of *in vivo* inhibition studies on different mycobacterial species.

Inhibitor	Bacterium	Inhibitory Effect	References
EZA	*M. tuberculosis* ^a^	Attenuates virulence, inhibits PhoPR	[36]
EZA	*M. avium* ^b^	Transport of eDNA and biofilm formation	[35]
Dithiocarbamate 12	*M. marinum* ^b^	^c^ Impairs grow of bacterium in the larvae	[26]

^a^ Mycobacterium tuberculosis complex. ^b^ Nontuberculous mycobacteria. ^c^ In zebrafish larval model.

## Abbreviations

CA	carbonic anhydrase
Mtb	*Mycobacterium tuberculosis*
MIC	minimal inhibitory concentration
CAI	carbonic anhydrase inhibitor
TB	tuberculosis
MTC	Mycobacterium tuberculosis complex
NTM	nontuberculous mycobacteria
EZA	ethoxzolamide
DTCs	dithiocarbamates
ATZ	acetazolamide
DZA	dorzolamide
eDNA	extracellular DNA

## References

[1] Stahl D.A., Urbance J.W. (1990). The division between fast- and slow-growing species corresponds to natural relationships among the mycobacteria. J. Bacteriol..

[2] Kim C.J., Kim N.H., Song K.H., Choe P.G., Kim E.S., Park S.W., Kim H.B., Kim N.J., Kim E.C., Park W.B. (2013). Differentiating rapid- and slow-growing mycobacteria by difference in time to growth detection in liquid media. Diagn. Microbiol. Infect. Dis..

[3] Sinha P., Gupta A., Prakash P., Anupurba S., Tripathi R., Srivastava G.N. (2016). Differentiation of Mycobacterium tuberculosis complex from non-tubercular mycobacteria by nested multiplex PCR targeting IS6110, MTP40 and 32kD alpha antigen encoding gene fragments. BMC Infect. Dis..

[4] WHO (2018). Global Tuberculosis Report 2018.

[5] WHO (2017). World Health Organization Lepracy: Fact Sheet on Leprosy.

[6] Daley C.L., Griffith D.E. (2010). Pulmonary non-tuberculous mycobacterial infections. Int. J. Tuberc. Lung Dis..

[7] Adrados B., Julián E., Codony F., Torrents E., Luquin M., Morató J. (2011). Prevalence and concentration of non-tuberculous mycobacteria in cooling towers by means of quantitative PCR: A prospective study. Curr. Microbiol..

[8] King H.C., Khera-Butler T., James P., Oakley B.B., Erenso G., Aseffa A., Knight R., Wellington E.M., Courtenay O. (2017). Environmental reservoirs of pathogenic mycobacteria across the Ethiopian biogeographical landscape. PLoS ONE.

[9] Falkinham J.O. (2013). Ecology of nontuberculous mycobacteria--where do human infections come from?. Semin. Respir. Crit. Care Med..

[10] Gopinath K., Singh S. (2010). Non-tuberculous mycobacteria in TB-endemic countries: Are we neglecting the danger?. PLoS Negl. Trop. Dis..

[11] Johnson M.M., Odell J.A. (2014). Nontuberculous mycobacterial pulmonary infections. J. Thorac. Dis..

[12] Faria S., Joao I., Jordao L. (2015). General Overview on Nontuberculous Mycobacteria, Biofilms, and Human Infection. J. Pathog..

[13] Misch E.A., Saddler C., Davis J.M. (2018). Skin and Soft Tissue Infections Due to Nontuberculous Mycobacteria. Curr. Infect. Dis. Rep..

[14] Al-Ghafli H., Al-Hajoj S. (2017). Nontuberculous Mycobacteria in Saudi Arabia and Gulf Countries: A Review. Can. Respir. J..

[15] Nishiuchi Y., Iwamoto T., Maruyama F. (2017). Infection Sources of a Common Non-tuberculous Mycobacterial Pathogen, *Mycobacterium avium* Complex. Front. Med..

[16] Fedrizzi T., Meehan C.J., Grottola A., Giacobazzi E., Fregni Serpini G., Tagliazucchi S., Fabio A., Bettua C., Bertorelli R., De Sanctis V. (2017). Genomic characterization of Nontuberculous Mycobacteria. Sci. Rep..

[17] Stinear T.P., Seemann T., Harrison P.F., Jenkin G.A., Davies J.K., Johnson P.D., Abdellah Z., Arrowsmith C., Chillingworth T., Churcher C. (2008). Insights from the complete genome sequence of *Mycobacterium marinum* on the evolution of *Mycobacterium tuberculosis*. Genome Res..

[18] Cole S.T., Brosch R., Parkhill J., Garnier T., Churcher C., Harris D., Gordon S.V., Eiglmeier K., Gas S., Barry C.E. (1998). Deciphering the biology of Mycobacterium tuberculosis from the complete genome sequence. Nature.

[19] Vissa V.D., Brennan P.J. (2001). The genome of Mycobacterium leprae: A minimal mycobacterial gene set. Genome Biol..

[20] Cole S.T., Eiglmeier K., Parkhill J., James K.D., Thomson N.R., Wheeler P.R., Honoré N., Garnier T., Churcher C., Harris D. (2001). Massive gene decay in the leprosy bacillus. Nature.

[21] Showalter H.D., Denny W.A. (2008). A roadmap for drug discovery and its translation to small molecule agents in clinical development for tuberculosis treatment. Tuberculosis.

[22] Covarrubias A.S., Larsson A.M., Högbom M., Lindberg J., Bergfors T., Björkelid C., Mowbray S.L., Unge T., Jones T.A. (2005). Structure and function of carbonic anhydrases from Mycobacterium tuberculosis. J. Biol. Chem..

[23] Covarrubias A.S., Bergfors T., Jones T.A., Högbom M. (2006). Structural mechanics of the pH-dependent activity of beta-carbonic anhydrase from Mycobacterium tuberculosis. J. Biol. Chem..

[24] Winum J.-Y., Stephan K., Scozzafava A., Montero J.-L., Supuran C.T. (2008). Targeting bacterial metalloenzymes: A new strategy for the development of anti-infective agents. Anti-Infect. Agents Med. Chem..

[25] Kapopoulou A., Lew J.M., Cole S.T. (2011). The MycoBrowser portal: A comprehensive and manually annotated resource for mycobacterial genomes. Tuberculosis.

[26] Aspatwar A., Hammarén M., Koskinen S., Luukinen B., Barker H., Carta F., Supuran C.T., Parikka M., Parkkila S. (2017). beta-CA-specific inhibitor dithiocarbamate Fc14-584B: A novel antimycobacterial agent with potential to treat drug-resistant tuberculosis. J. Enzyme Inhib. Med. Chem..

[27] Minakuchi T., Nishimori I., Vullo D., Scozzafava A., Supuran C.T. (2009). Molecular cloning, characterization, and inhibition studies of the Rv1284 beta-carbonic anhydrase from *Mycobacterium tuberculosis* with sulfonamides and a sulfamate. J. Med. Chem..

[28] Nishimori I., Minakuchi T., Vullo D., Scozzafava A., Innocenti A., Supuran C.T. (2009). Carbonic anhydrase inhibitors. Cloning, characterization, and inhibition studies of a new beta-carbonic anhydrase from *Mycobacterium tuberculosis*. J. Med. Chem..

[29] Carta F., Maresca A., Covarrubias A.S., Mowbray S.L., Jones T.A., Supuran C.T. (2009). Carbonic anhydrase inhibitors. Characterization and inhibition studies of the most active beta-carbonic anhydrase from *Mycobacterium tuberculosis*, Rv3588c. Bioorg. Med. Chem. Lett..

[30] Nishimori I., Minakuchi T., Morimoto K., Sano S., Onishi S., Takeuchi H., Vullo D., Scozzafava A., Supuran C.T. (2006). Carbonic anhydrase inhibitors: DNA cloning and inhibition studies of the alpha-carbonic anhydrase from Helicobacter pylori, a new target for developing sulfonamide and sulfamate gastric drugs. J. Med. Chem..

[31] Supuran C.T. (2008). Carbonic anhydrases: Novel therapeutic applications for inhibitors and activators. Nat. Rev. Drug Discov..

[32] Supuran C.T. (2016). Structure and function of carbonic anhydrases. Biochem. J..

[33] Sassetti C.M., Boyd D.H., Rubin E.J. (2003). Genes required for mycobacterial growth defined by high density mutagenesis. Mol. Microbiol..

[34] Sassetti C.M., Rubin E.J. (2003). Genetic requirements for mycobacterial survival during infection. Proc. Natl. Acad. Sci. USA.

[35] Rose S.J., Bermudez L.E. (2016). Identification of Bicarbonate as a Trigger and Genes Involved with Extracellular DNA Export in Mycobacterial Biofilms. MBio.

[36] Johnson B.K., Colvin C.J., Needle D.B., Mba Medie F., Champion P.A., Abramovitch R.B. (2015). The Carbonic Anhydrase Inhibitor Ethoxzolamide Inhibits the Mycobacterium tuberculosis PhoPR Regulon and Esx-1 Secretion and Attenuates Virulence. Antimicrob Agents Chemother..

[37] Nishimori I., Minakuchi T., Maresca A., Carta F., Scozzafava A., Supuran C.T. (2010). The beta-carbonic anhydrases from Mycobacterium tuberculosis as drug targets. Curr. Pharm. Des..

[38] Güzel O., Maresca A., Scozzafava A., Salman A., Balaban A.T., Supuran C.T. (2009). Discovery of low nanomolar and subnanomolar inhibitors of the mycobacterial beta-carbonic anhydrases Rv1284 and Rv3273. J. Med. Chem..

[39] Maresca A., Carta F., Vullo D., Scozzafava A., Supuran C.T. (2009). Carbonic anhydrase inhibitors. Inhibition of the Rv1284 and Rv3273 beta-carbonic anhydrases from Mycobacterium tuberculosis with diazenylbenzenesulfonamides. Bioorg. Med. Chem. Lett..

[40] Carta F., Maresca A., Scozzafava A., Vullo D., Supuran C.T. (2009). Carbonic anhydrase inhibitors. Diazenylbenzenesulfonamides are potent and selective inhibitors of the tumor-associated isozymes IX and XII over the cytosolic isoforms I and II. Bioorg. Med. Chem..

[41] Pacchiano F., Carta F., Vullo D., Scozzafava A., Supuran C.T. (2011). Inhibition of beta-carbonic anhydrases with ureido-substituted benzenesulfonamides. Bioorg. Med. Chem. Lett..

[42] Maresca A., Scozzafava A., Vullo D., Supuran C.T. (2013). Dihalogenated sulfanilamides and benzolamides are effective inhibitors of the three beta-class carbonic anhydrases from Mycobacterium tuberculosis. J. Enzym. Inhib. Med. Chem..

[43] Ceruso M., Vullo D., Scozzafava A., Supuran C.T. (2014). Sulfonamides incorporating fluorine and 1,3,5-triazine moieties are effective inhibitors of three beta-class carbonic anhydrases from Mycobacterium tuberculosis. J. Enzym. Inhib. Med. Chem..

[44] Wani T.V., Bua S., Khude P.S., Chowdhary A.H., Supuran C.T., Toraskar M.P. (2018). Evaluation of sulphonamide derivatives acting as inhibitors of human carbonic anhydrase isoforms I, II and Mycobacterium tuberculosis beta-class enzyme Rv3273. J. Enzym. Inhib. Med. Chem..

[45] Maresca A., Carta F., Vullo D., Supuran C.T. (2013). Dithiocarbamates strongly inhibit the beta-class carbonic anhydrases from Mycobacterium tuberculosis. J. Enzym. Inhib. Med. Chem..

[46] Davis R.A., Hofmann A., Osman A., Hall R.A., Mühlschlegel F.A., Vullo D., Innocenti A., Supuran C.T., Poulsen S.A. (2011). Natural product-based phenols as novel probes for mycobacterial and fungal carbonic anhydrases. J. Med. Chem..

[47] Cau Y., Mori M., Supuran C.T., Botta M. (2016). Mycobacterial carbonic anhydrase inhibition with phenolic acids and esters: Kinetic and computational investigations. Org. Biomol. Chem..

[48] Buchieri M.V., Riafrecha L.E., Rodríguez O.M., Vullo D., Morbidoni H.R., Supuran C.T., Colinas P.A. (2013). Inhibition of the beta-carbonic anhydrases from Mycobacterium tuberculosis with C-cinnamoyl glycosides: Identification of the first inhibitor with anti-mycobacterial activity. Bioorg. Med. Chem. Lett..

[49] Maresca A., Vullo D., Scozzafava A., Manole G., Supuran C.T. (2013). Inhibition of the beta-class carbonic anhydrases from Mycobacterium tuberculosis with carboxylic acids. J. Enzym. Inhib. Med. Chem..

[50] Scozzafava A., Mastrolorenzo A., Supuran C.T. (2001). Antimycobacterial activity of 9-sulfonylated/sulfenylated-6-mercaptopurine derivatives. Bioorg. Med. Chem. Lett..

[51] Rose S.J., Babrak L.M., Bermudez L.E. (2015). Mycobacterium avium Possesses Extracellular DNA that Contributes to Biofilm Formation, Structural Integrity, and Tolerance to Antibiotics. PLoS ONE.

[52] Johnson B.K., Abramovitch R.B. (2017). Small Molecules That Sabotage Bacterial Virulence. Trends Pharmacol. Sci..

